# WGCNA Analysis of Salt-Responsive Core Transcriptome Identifies Novel Hub Genes in Rice

**DOI:** 10.3390/genes10090719

**Published:** 2019-09-17

**Authors:** Mingdong Zhu, Hongjun Xie, Xiangjin Wei, Komivi Dossa, Yaying Yu, Suozhen Hui, Guohua Tang, Xiaoshan Zeng, Yinghong Yu, Peisong Hu, Jianlong Wang

**Affiliations:** 1 Hunan Agricultural University, Changsha 410128, China; uhz_uhz@hotmail.com (M.Z.); villy816@163.com (Y.Y.); huisuozhen@126.com (S.H.); 2 Hunan Rice Research Institute, Changsha 410125, China; xhj1110@126.com (H.X.); tgh579@hotmail.com (G.T.); zengxiaoshan11@outlook.com (X.Z.); 3 China National Rice Research Institute, Hangzhou 311401, China; weixiangjin@caas.cn (X.W.); hupeisong@caas.cn (P.H.); 4 Wuhan Benagen Tech Solutions Company Limited, Wuhan 430070, China; komiri.dossa@ucad.edu.sn; 5 Hunan Academy of Agricultural Sciences, Changsha 410125, China; yyh30678@163.com

**Keywords:** salt stress, transcriptome, weighted gene co-expression network analysis (WGCNA), co-expressed genes, network analysis, rice

## Abstract

Rice, being a major staple food crop and sensitive to salinity conditions, bears heavy yield losses due to saline soil. Although some salt responsive genes have been identified in rice, their applications in developing salt tolerant cultivars have resulted in limited achievements. Herein, we used bioinformatic approaches to perform a meta-analysis of three transcriptome datasets from salinity and control conditions in order to reveal novel genes and the molecular pathways underlying rice response to salt. From a total of 28,432 expressed genes, we identify 457 core differentially expressed genes (DEGs) constitutively responding to salt, regardless of the stress duration, genotype, or the tissue. Gene co-expression analysis divided the core DEGs into three different modules, each of them contributing to salt response in a unique metabolic pathway. Gene ontology and Kyoto Encyclopedia of Genes and Genomes (KEGG) analyses highlighted key biological processes and metabolic pathways involved in the salt response. We identified important novel hub genes encoding proteins of different families including CAM, DUF630/632, DUF581, CHL27, PP2-13, LEA4-5, and transcription factors, which could be functionally characterized using reverse genetic experiments. This novel repertoire of candidate genes related to salt response in rice will be useful for engineering salt tolerant varieties.

## 1. Introduction

Salinity stress is one of the leading abiotic stresses that challenge the sustainability of crop production [1]. A high level of salt in soil inhibits plant growth, induces wilting and the death of plants. Salt stress causes ion toxicity by Na+ and Cl- ions leakage, which leads to disruption of the cell membrane, inhibition of protein synthesis, and alteration of enzyme activity [2]. Importantly, salinity also causes a reduction in photosynthesis, resulting in chlorosis and programmed cell death [3]. Different classes of genes including phosphatases, kinases, hormones, and transcription factors play significant roles in salt stress responses [4]. In recent years, various genes conferring salt tolerance in plants have been identified and shown to be involved in transcription, signal transduction, ion transport, and metabolic pathways [5,6]. 

Rice is a staple food crop of half of the world population and a genomic model crop of the monocot family. Rice production is supposed to be increased by 0.6% to 0.9% every year until 2050 in order to feed a rapidly increasing population [7]. Rice, being sensitive to salinity conditions, bears heavy yield losses due to saline soil [8]. Extensive omics data have been generated and applied in various studies towards rice yield and quality improvement. In particular, transcriptome sequencing and computational approaches have greatly facilitated rice molecular research [9]. Some important salt responsive genes such as *OsSOS1*, *OsNHX1*, *OsHKT2*;1, *OsCAX1*, *OsAKT1*, *OsKCO1*, *OsTPC1*, *OsCLC1*, and *OsNRT1;2* have been discovered in rice but so far, very limited achievements have been made to develop salt-tolerant cultivars [10]. In fact, plant response to stress is a coordinated action of multiple stress responsive genes, interacting with other components of stress signal transduction pathways [11]. Therefore, there are still large numbers of unidentified genes with high potential to improve salt tolerance in rice. In recent years with the development of new high-throughput technologies such as RNA sequencing (RNA-seq) and data analysis methods, the functional characteristics of thousands of genes can be investigated systematically [12]. In rice, various RNA-seq studies have been conducted to explore the differentially expressed genes under salinity stress [13,14,15,16,17]. These datasets represent valuable genomic resources to perform meta-analysis to identify the core-conserved genes modulating salt responses in rice, regardless of the stress intensity, genotype, environment, etc. 

One important method to understand the gene function and gene association from genome-wide expression is the co-expression network analysis [18,19]. The co-expression network approach constructs the network of genes with co-activation across a group of samples. Nowadays, weighted gene co-expression network analysis (WGCNA) is the most commonly used system biology approach to identify the pattern of correlations among genes [20]. It is useful for the identification of the modules of co-expressed genes, their correlation with external traits, and the pinpointing of key hub genes. It has been widely applied to detect the co-expressed genes responsive to stress and cell wall organization in cotton [21], salt stress response in Arabidopsis and rice [22,23], and the biotic stress response in Arabidopsis [24].

In the present study, we re-analyzed three diverse salt-stress transcriptomic datasets in rice and identified the core salt-stress responsive genes. Further, by applying WGCNA, we identified three functional modules and several biological and metabolic pathways that are involved in rice response to salt. Finally, we proposed various putative novel salt stress-responsive uncharacterized genes that can be harnessed to improve salt tolerance in rice.

## 2. Materials and Methods 

### 2.1. Plant Material and Growth Conditions

The seeds of japonica rice cultivar “Hunan” were collected from Hunan Rice Research Institute, Changsha, China. The experiment was conducted in controlled environment of a greenhouse. The seeds were surface sterilized with 1% NaOCl solution to remove the contaminants. Sterilized seeds were immersed in water at 37 °C for two days followed by germination at 30 °C with a photoperiod of 16 h (light)/8 h (dark) and a relative humidity set at 70%. Seedlings were grown for a week in 2000 ml boxes containing a ½ strength Hoagland nutrient solution. Then, the salt stress condition was applied by adding to the nutrient solution 200 mM NaCl solution in one step and then the whole plant (shoot + root) was harvested after 0, 3, 6, and 12 h time period later. The control seedlings were maintained in a nutrient solution without salt treatment and samples were collected in parallel. Three biological replicates were maintained for control and salt treatment for each time point. 

### 2.2. RNA Isolation and qRT-PCR Gene Expression Analysis

Plant total RNA from control and salt-treated samples was isolated using an RNA extraction kit (Tiangen, Beijing, China), and the first-strand cDNA was synthesized from 2 µg of RNA by reverse transcriptase (Invitrogen, Carlsbad, CA, USA), and then diluted (1:4) for use in qRT-PCR with SYBR Premix ExTaq Mix (Takara, Dalian, Liaoning, China) in a total volume of 20 µL. Reactions were performed in a LightCycler 480 thermal cycler (Roche, Basel, Switzerland), following the manufacturer’s instructions. Three biological replicates were analyzed for each sample, and the expression level was normalized to that of the *rice Actin-1* gene (*LOC4333919*), which is stably and constitutively expressed in rice tissues and under various stress conditions [25]. The primer sequences used in this study are given in Table S1.

### 2.3. Data Acquisition and RNA-Seq Analysis

RNA sequence data for control and salt-treated japonica rice cultivars were downloaded from the National Center for Biotechnology Information (NCBI) Sequence Read Archive (SRA) (https://www.ncbi.nlm.nih.gov/sra). After quality control, only datasets with high quality (clean data with at least 90% of bases scoring Q30 and above) were kept and their SRA accessions and information are listed in Table 1. The rice reference genome and gene model annotation files (MSU7.0) were downloaded from the JGI database directly. We mapped the reads to the *Oryza sativa* L. japonica. cv. Nipponbare genome using STAR (2.5.1b) [26] and then “Trimmed Mean of M-values” (TMM) normalized fragments per kilobase of transcript per million fragments mapped (FPKM) values were used to estimate the gene expression level [27]. A stringent criterion (fold-change ≥ 2 and *q*_value ≤ 0.05, with a significant false discovery rate-adjusted *p* value (FDR) < 0.05) was used to screen out the differentially expressed genes (DEGs) between each set of compared samples by the edgeR software [28]. Significance of the overlap between DEGs in the three datasets was estimated using the hypergeometric test computed with the “*phyper*” function in the R software (http://www.r-project.org) with a Bonferroni correction of the *p* values. 

### 2.4. Gene Ontology and KEGG Analysis of the Core DEGs

Salt responsive core DEGs were identified by comparing all DEGs among different datasets using Venn diagram analysis. These genes were then subjected to Gene Ontology (GO) and Kyoto Encyclopedia of Genes and Genomes (KEGG) significant enrichment analysis to identify the enriched biological processes and metabolic pathways involved in salt tolerance. GO and KEGG enrichment was analyzed using clusterProfiler. The heatmap was exhibited using “heat map” R-package. The information of transcription factor families was downloaded from Plant TFDB (planttfdb.cbi.pku.edu.cn/).

### 2.5. Weighted Gene Co-Expression Network Analysis (WGCNA)

Gene co-expression networks were constructed using the WGCNA package in the R software. The core DEGs were further divided into three modules using WGCNA and correlation of each module with salt stress was calculated. Module-trait associations were estimated using the correlation between the module eigengene and salt/control treatments. Network visualization for each module was performed using the Cytoscape software version 3.6.1 with a cut-off of the weight parameter obtained from the WGCNA set at 0.3. [29]. The gene co-expression network is a scale-free weighted gene network with multiple nodes connected to different nodes via edges. Each node represents a gene, which is connected to a different number of genes. The gene which is connected to a greater number of genes is denoted with a bigger size and is more important for its interaction with a large number of genes. 

## 3. Results

### 3.1. Identification of the Salt-Responsive Core DEGs in Rice

In this study, we analyzed the global gene expression profiles of japonica rice cultivars for salt stress response using different datasets, namely, SRP114666, SRP076274, and SRP083700. The details of these datasets including treatment, tissue, and accession numbers are given in Table 1. From a total of 28,432 expressed genes among the different datasets, 15,596 unique differentially expressed genes (DEGs) were identified between control and stressed samples in the different datasets (Table S2).

**Figure 1:**
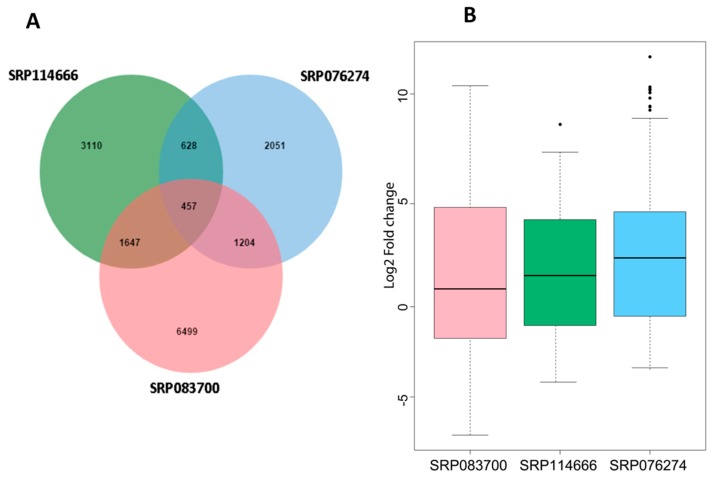
Identification of salt responsive core differentially expressed genes (DEGs) in rice. (**A**) Venn diagram showing specific and common salt responsive DEGs among the RNA-seq datasets used in this study; (**B**) Expression profiles of the core salt responsive DEGs based on fragments per kilobase of transcript per million fragments mapped (FPKM) values.

In order to identify the salt responsive core DEGs, we cross-compared the DEGs among the three different datasets, which resulted in 457 core DEGs that are common in rice, independently of the tissue type, genotype, and the salt stress duration/intensity. These core DEGs were statistically significant based on the hypergrometric test (*p* < 0.001) There were 628 common DEGs among SRP114666 and SRP076274; 1204 common DEGs among SRP076274 and SRP083700; 1,647 common DEGs among SRP114666 and SRP083700 (Figure 1A). The expression fold changes between control and stress treatments of these 457 core DEGs greatly varied among datasets but were mainly up-regulated under stress, confirming that these core DEGs are responsive to salt (Figure 1B). The thousands of genes specific to each dataset may be caused by differences in salt tolerance levels of the genotypes, their genetic make-up, the sampled tissues, salt stress treatments, and growth conditions.

**Figure 2:**
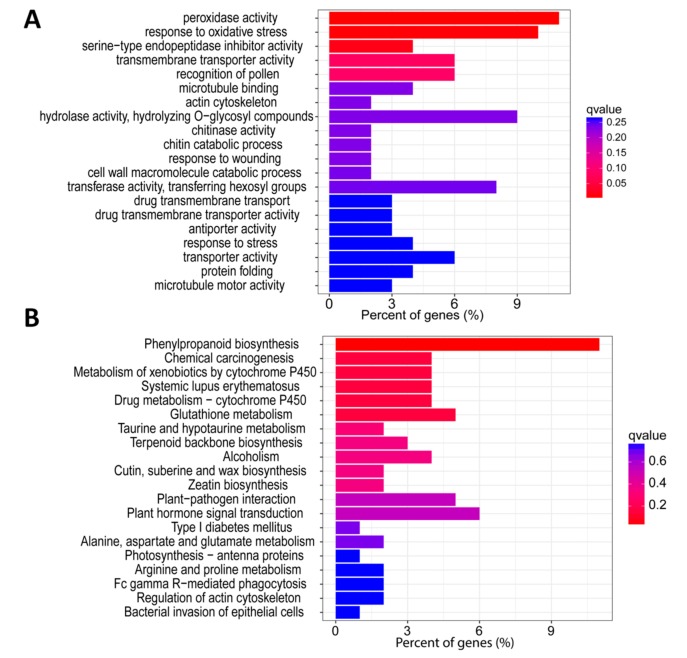
(**A**) Gene ontology and (**B**) Kyoto Encyclopedia of Genes and Genomes (KEGG) enrichment analyses of core salt responsive DEGs.

We then characterized these 457 core DEGs to get insight into their contributing molecular pathways. To achieve this, we first performed gene ontology (GO) to identify the significantly enriched biological processes contributed by these DEGs. GO analysis unveiled that “peroxidase activity” was the most enriched biological process followed by “response to stress” with the *q*-value lower than 0.1, suggesting that peroxidase genes are involved in salt response by regulating the antioxidant activity (Figure 2A). Next, we performed Kyoto Encyclopedia of genes and genomes (KEGG) analysis to identify enriched pathways contributed by the 457 core salt responsive DEGs. Phenylpropanoid biosynthesis was the most significantly enriched KEGG pathway followed by glutathione metabolism, indicating that phenylpropanoid and glutathione play key roles in salt response in rice (Figure 2B). 

### 3.2. WGCNA of the Salt-Responsive Core Genome

**Figure 3:**
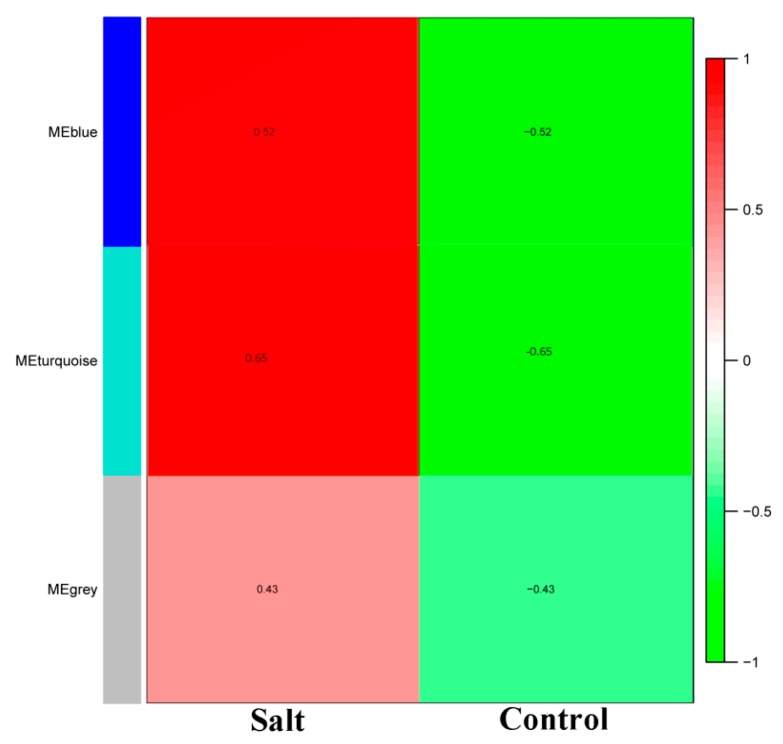
Matrix showing Module-Trait Relationships (MTRs) of different modules under control and salt stress. The numbers represent the Pearson correlation coefficients. Positive correlation is colored in red while negative correlation is colored in green.

**Figure 4:**
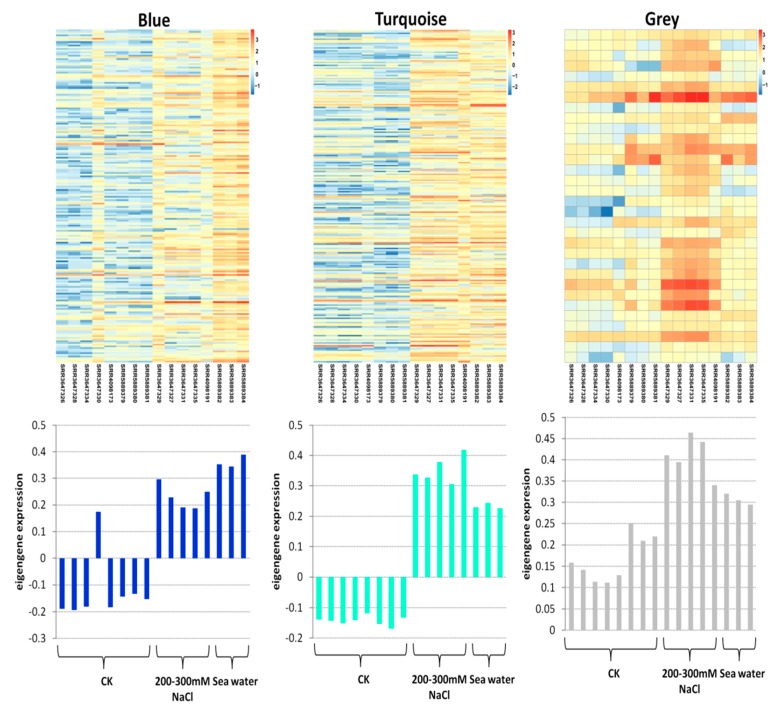
Expression pattern of the genes and eigengenes of each module. The heatmap was plotted using the log_10_ FPKM values.

In order to identify the different co-expressed modules under salinity stress in rice, we conducted a WGCNA on the 457 core DEGs. We successfully obtained three modules: Blue, grey and turquoise (Figure 3). The blue module contained 196 DEGs, the grey module contained 32 DEGs and turquoise module contained 229 DEGs (Table S3). All the modules had a positive correlation (*r* = 0.52, *r* = 0.43 and *r* = 0.65 for blue, grey, and turquoise, respectively) with salt stress, suggesting that genes in these modules positively regulate salt tolerance in rice. Thus, these genes should be up-regulated under salt stress to achieve salt tolerance. To further understand the particularity of each co-expressed module with respect to their expression patterns in the different datasets, we plotted the log_10_ FPKM values of the genes belonging to each module along with the eigengene expression values (Figure 4). We observed that in all the detected modules, the gene expression levels were higher under salt stress than in control condition, confirming the positive correlations observed earlier. The blue module genes appeared to be more responsive to sea water treatment than 200–300 mM NaCl treatments. Since seawater contains approximately 600 mM NaCl, we deduce that blue module genes are more responsive to high salt concentration. The turquoise module genes displayed the opposite trend with more induction under 200–300 mM NaCl treatments than under seawater treatment. Finally, we found that the grey module genes were particularly highly expressed in the control condition and the magnitude of induction under salt stress was weak as compared to the blue and turquoise genes.

### 3.3. GO and KEGG Enrichment Analysis of the Detected Co-Expressed Modules

To reveal the specific functions played by each co-expressed module, we performed GO and KEGG analysis of DEGs from each module separately. GO analysis indicated that “response to oxidative stress” and “peroxidase activity” were the most significantly enriched biological processes in the blue module (Figure S1A). This suggests that genes in the blue module are involved in tolerance to oxidative stress perhaps via scavenging of reactive oxygen species (ROS) such as hydrogen peroxide, superoxide radicals, etc. KEGG analysis indicated “phenylpropanoid biosynthesis” and “plant hormone signal transduction” as the most significantly enriched metabolic pathway, suggesting that genes in blue module contribute to salt tolerance via regulating phenylpropanoid related metabolites and plant hormones (Figure S1B). Regarding grey module, GO analysis identified “iron ion binding” as the most significantly enriched biological process which means that most of the genes in this module contribute to salt tolerance by regulating iron ion binding (Figure S2A). Curiously, KEGG analysis of grey module DEGs displayed various metabolic pathways that were equally enriched, suggesting that they are engaged in diverse molecular pathways (Figure S2B). Concerning the turquoise module, GO analysis displayed “ADP binding” as the most significantly enriched biological process, denoting that regulation of energy metabolism is essential for salinity tolerance (Figure S3A). Notably, KEGG analysis of turquoise module genes identified “phenylpropanoid biosynthesis” and “gluthatione metabolism” as the most significantly enriched metabolic pathway showing that genes in this module regulate phenylpropanoid related metabolites but also the antioxidant gluthatione in response to salt stress (Figure S3B).

### 3.4. Networks Displaying Relationships among Genes within Co-Expressed Modules

**Figure 5:**
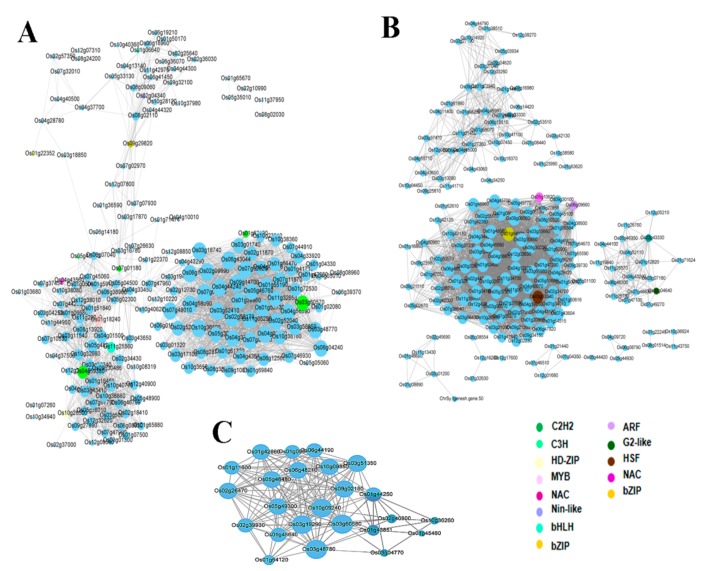
Co-expression network analysis of blue (**A**), turquoise (**B**) and grey (**C**) modules. The size of node circle is positively correlated with the number of the interacting genes.

We constructed the network of the detected co-expressed modules with the aim to identify key hub genes. Genes encoding transcription factors (TFs) are represented with different node colors except sky blue. The size of the node circle is positively correlated with the number of genes that it partners in interaction. Genes in the blue module were divided into three clusters, each having a network of a different number of genes (Figure 5). In gene networks, a smaller subset of genes (hub genes) interacts with many other genes and it is suggested that they are three times more likely to be essential than genes with fewer interaction partners [30]. In the present study, we identified 15 hub genes from the three modules encoding different proteins including carboxyesterase, calmodulin binding protein, DNA binding protein, LEA4-5, low temperature and salt responsive proteins (Table 2). Among the 15 hub genes, we report two unknown genes (LOC_Os05g27340, LOC_Os01g72009) and two proteins of unknown domains DUF630/632 (LOC_Os02g43770) and DUF581 (LOC_Os09g20240). Transcription factors (TF) are well known to play a crucial role in abiotic stress tolerance in plants by regulating the expression of stress-responsive genes [31,32]. Therefore, we searched for the TFs within each module detected by WGCNA in this study. TFs in the blue module include C2H2-type zinc finger (LOC_Os03g60570, LOC_Os01g62190, LOC_Os04g59380, and LOC_Os07g01180), basic helix–loop–helix, bHLH (LOC_Os11g25560), myeloblastosis, MYB (LOC_Os01g18240), basic leucine-zipper, bZIP (LOC_Os09g29820), NAC (LOC_Os04g43560) and plant regulator RWP-RK family protein (LOC_Os02g04340). Each of these TFs interacts with several target genes and may regulate their expression (Figure 5A). TF genes detected in the turquoise module are bZIP (LOC_Os01g64000), HSF (LOC_Os03g53340), NAC (LOC_Os05g10620), ARF (LOC_Os06g09660), homeobox (LOC_Os02g43330), and MYB (LOC_Os02g04640) (Figure 5B). Notably, the grey module does not have any gene encoding TF (Figure 5C).

### 3.5. qRT-PCR Validation of Selected Genes from Each Module under Temporal Salt Stress

**Figure 6:**
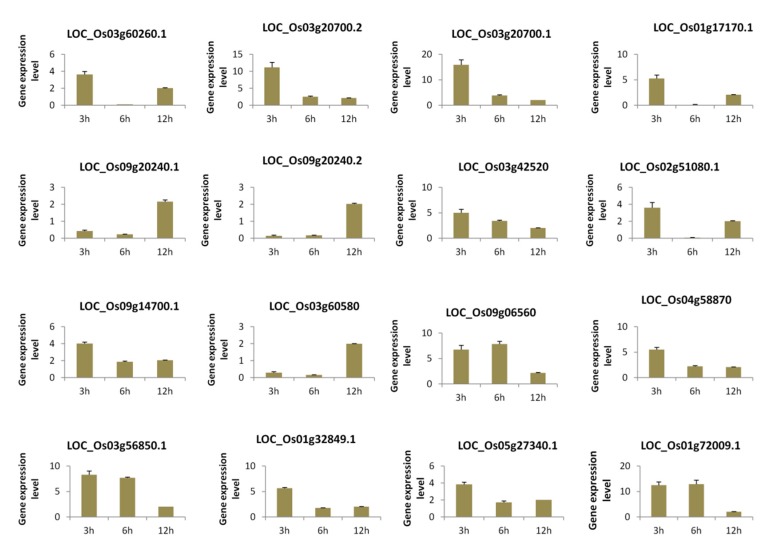
qRT-PCR analysis of selected genes from the rice core salt stress responsive genes under temporal salt stress compared to the control condition. Data are from three biological replicates and three technical replicates. The differential expression analysis was conducted based on the 2^−ΔΔct^ method.

In order to experimentally confirm the results of our computational analysis, we selected 16 genes mainly the hub genes from the three modules and performed a quantitative reverse-transcription PCR (qRT-PCR) analysis of their expression levels after 3 h, 6 h, and 12 h salt stress treatments in an independent rice cultivar “Hunan”. The results showed that the expression levels of all the selected genes were significantly changed at each time point under salt stress as compared to control, demonstrating that the genes were all responsive to salt (Figure 6).

## 4. Discussion

Salt stress is major abiotic stress reducing production in major cereal crops including maize [33], barley [34], wheat [35], and rice [36]. Rice is a very sensitive crop to salinity stress and its yield and productivity are critically impaired due to highly increasing salinity levels in agricultural soil. Thus, it is important to understand the molecular mechanisms underlying salt response in rice. In recent years, transcriptomic data has opened up the doors to analyze and unravel the molecular mechanism and biological processes involved in abiotic and biotic stress response in plants. RNA sequencing (RNA-seq) analysis is a critical, easy, rapid, and economical approach of transcriptome studies [37]. Different stress responsive genes have been identified by RNA-seq analysis and their expression under salinity stress already been clarified in various plants including *Glycine max* [38], rice [39], sweet potato [40], and wild barley [41]. RNA-seq generates a bundle of information for a target phenotype or stress; however, resourceful utilization of this data has been a bottleneck. Recently, availability of several bioinformatics and statistical tools have helped plant scientists to pinpoint key biological processes and metabolic pathways involved in biotic or abiotic stress tolerance, through meta-analysis of large RNA-seq datasets [42].

The present study was aimed at understanding the central players of salt response in rice through an analysis of three RNA-seq datasets. We unraveled 457 genes constantly altered under salt stress in all datasets which may be essential for rice salt responses since they were not specific to a tissue type, genotype, or stress intensity. Moreover, a subset of these genes was validated through qRT-PCR in an independent rice cultivar, proving that the salt responsive core DEGs detected in the present study is common in rice. GO and KEGG enrichment analyses of the core DEGs unveiled various biological pathways contributed by these genes under salt stress. “Peroxidase activity” was the most enriched biological process under salt response in rice (Figure 2A). During various environmental stimuli, plants generate and accumulate significant level of reactive oxygen species (ROS) such as superoxide anions (O-2O2-), hydrogen peroxide (H_2_O_2_), hydroxyl radicals (OH), and singlet oxygen (^1^O_2_) [43]. High accumulation of ROS leads to oxidative damage to cellular membranes (lipid peroxidation), proteins, RNA, and DNA, resulting in irreversible cellular damage and even cell death [44]. Plants have strong ROS scavenging enzymes and antioxidants to neutralize ROS. Peroxidase is one of the scavenging enzymes and is reported to have important role in enhancing tolerance against various stresses including salinity [45]. Earlier studies have reported hydrogen peroxide (H_2_O_2_) accumulation during salt stress treatment in rice [46]. Thus, based on the result of our study, an increase activity of peroxidase genes may lead to salt tolerance in rice. Many genes also contributed to “phenylpropanoid pathway”, suggesting that during salt stress, regulation of phenylpropanoid metabolites helps rice to combat salt damage. Various enzymes involved in phenylpropanoid pathway such as Phenyl ammonium Lyase (PAL) serve as biochemical markers for stress conditions [47]. Earlier reports suggested that higher PAL levels are directly related to increased tolerance to environmental stress [48]. Ref. [49] found that increased NaCl concentrations enhance PAL enzyme activity in *Jatropha curcas* seedlings. PAL and flavonoid pathways related structural genes are considered as two critical defense signaling cascades during environmental stress in plants [50]. Ref. [51] reported that activation of PAL as a key component of the antioxidant system in salt-challenged maize is a promising target for maize salt resistance engineering. We also found “iron ion binding” as a significantly enriched biological process. Iron (Fe) is one of the crucial micronutrients for plant growth and development. During the whole life cycle of a plant, iron performs most of the major functions from chlorophyll biosynthesis to energy transfer [52]. It has been reported that salinity inhibits the deposition and distribution of nutrients in the plants [53,54]. The most frequent feature during salinity is chlorosis due to a limited supply of Fe to plants. Ref. [55] showed the inhibitory effect of salinity on the accumulation of Fe content in the shoots of peas. We speculate that manipulation of target core DEGs will favor optimum Fe supply to rice plants under salinity stress. Another important enriched metabolic pathway was “starch and sugar metabolism”. Starch is an important molecule that mediates plant responses to abiotic stresses, including drought, salinity and extreme temperatures [56,57]. When the photosynthesis is potentially limited, plants remobilize starch to provide energy and carbon. Sugar metabolites are considered as osmoprotectants and compatible solutes to alleviate the negative effects of stress [58]. Sugar is another important carbohydrate and signaling molecule that also cross-talks with the ABA-dependent signaling mechanism to mitigate the stress damage [59]. Thus, we infer that the genes involved in starch and sugar metabolism regulate salt response in rice by inducing the carbohydrate metabolism.

WGCNA divided the core DEGs into three modules, each of them contributing to salt tolerance in a unique metabolic pathway (Figure 3). Importantly, we found that increasing the expression level of the core DEGs is beneficial for salt tolerance in rice. Network construction highlighted several hub genes predicted to play central roles in salt response in rice. Interestingly, most of these genes were unreported with regard to their involvement in salt response in rice. Several genes from the families of the hub genes (CAM, HSF, and DUF630/632) identified in blue module have been reported to regulate the abiotic stress response. For example, transgenic rice over-expressing the calmodulin gene *OsCam1–1* (*LOC_Os03g20370*) is more tolerant to salt stress than wild type [60]. Ref. [61] reported the role of DUF630/632 in controlling leaf rolling in rice, while its role under salinity tolerance is not yet confirmed. We also reported the hub TF *Heat Shock Factor c1* (*HSFc1*). Earlier reports showed that HSF (*OsHsfc1b*) regulates salt tolerance and development in rice [62]. The hub genes in grey module are pyridine nucleotide-disulphide oxidoreductase family proteins (*LOC_Os02g51080*, *LOC_Os03g20700*), dicarboxylate diiron protein, ACSF, CHL27 (*LOC_Os01g17170*) and Domain of unknown function DUF581 (*LOC_Os09g20240*). The DUF581 encoding gene was differentially expressed by hormones and environmental cues in Arabidopsis [63]. Pyridine nucleotide-disulphide oxidoreductases active site I is evolutionarily conserved in Glutathione Reductase (GR) in rice and Arabidopsis [64]. GR plays an important role in defending the plant from oxidative damage induced by various biotic and abiotic stressors [65]. In the turquoise module, we identified low temperature and salt responsive protein (*LOC_Os03g17790*), phloem protein 2-A13, (*LOC_Os04g48270*), and Late Embryogenesis Abundant 4-5 (*LOC_Os08g23870*). These genes might play preponderant functions for salt stress response in rice. *OsLEA3* gene overexpression in rice showed enhanced tolerance against drought and salinity [66]. *AtLEA4-5* is a member of the group 4 late embryogenesis abundant (LEA) proteins, which are involved in the tolerance of water deficits in Arabidopsis [67]. Dossa et al. [68] recently demonstrated that overexpression of a hub gene from the sesame core-abiotic stress responsive genes confer tolerance to multiple stresses in Arabidopsis. In this study, qRT-PCR analysis of the detected hub genes in an independent rice cultivar revealed that they were mostly up-regulated at different time points under salt stress, showing that increasing their expression levels would enhance salt tolerance in rice. Overall, the hub genes identified in the present study provide novel tools to be harnessed for engineering highly salt tolerant rice cultivars. 

Transcription factors have been well documented to play key roles in stress tolerance by regulating the stress-responsive gene expression [69]. We have identified C2H2 type zinc finger, MYB, bZIP, and NAC transcription factor family proteins as major regulators of the core salt responsive genes in rice. Several members of these TF families have been reported to regulate plant response to environmental stress including salinity [70,71]. MYB TFs are involved in plant development, secondary metabolism, signal transduction, and biotic and abiotic stress tolerance [72]. *OsMYB6* gene overexpression increased drought and salinity tolerance in rice [73]. bZIP TFs have been identified in different plants including Arabidopsis and rice [74,75]. They regulate the responses to biotic and abiotic stresses, including pathogen defense, hormone and sugar signaling, light response, and salt and drought tolerance [76]. *OsbZIP71* conferred salinity and drought tolerance in rice [77]. Stress-responsive NAC proteins have been reported as positive regulators of abiotic stress tolerance [78]. Hong et al. [78] identified a novel stress-responsive rice NAC gene, *ONAC022* and reported that its overexpression improves drought and salinity tolerance. Furthermore, rice plants overexpressing *STRESS-RESPONSIVE NAC1* (*SNAC1*) showed significantly improved drought and salt tolerance [79]. Altogether, we propose that these novel transcription factors could be functionally characterized using reverse genetic experiments.

## 5. Conclusions

In short, this study identified the core salt responsive genes and modules from diverse transcriptome datasets in rice. GO and KEGG analyses highlighted “peroxidase activity”, “phenylpropanoid pathways” and “plant hormone signal transduction”, as key biological processes and metabolic pathways involved in salt response in rice. Network analysis pinpointed several putative TFs from C2H2, Zinc-finger domain, homeobox domain, bZIP, and MYB families that could be important regulators of salt response in rice. Furthermore, hub genes identified in this study may be potential targets to engineer rice plants with improved salt tolerance. Additionally, this work lays a strong foundation for further investigation of the unknown proteins such as CHL27, PP2-13, DUF630/632, and DUF581 in rice and in other plants with reference to salt and other abiotic factors.

## Figures and Tables

**Table 1:** Overview of the RNA-seq datasets used in this study.

SRA Study	SRA Accession	Tissue	Treatment	Sequencing Platform	Cultivar	References
SRP076274	SRR3647326	Leaf	Unstressed-110d old seedling	Illumina HiSeq 2500	Nipponbare	-
SRP076274	SRR3647327	Leaf	Salt (200 mM)-110d old seedling	Illumina HiSeq 2500	Nipponbare	-
SRP076274	SRR3647328	Leaf	Unstressed-110d old seedling	Illumina HiSeq 2500	Nipponbare	-
SRP076274	SRR3647329	Leaf	Salt (200 mM)-110d old seedling	Illumina HiSeq 2500	Nipponbare	-
SRP076274	SRR3647330	Leaf	Unstressed-110d old seedling	Illumina HiSeq 2500	Nipponbare	-
SRP076274	SRR3647331	Leaf	Salt (200 mM)-110d old seedling	Illumina HiSeq 2500	Nipponbare	-
SRP076274	SRR3647334	Leaf	Unstressed-110d old seedling	Illumina HiSeq 2500	Nipponbare	-
SRP076274	SRR3647335	Leaf	Salt (200 mM)-110d old seedling	Illumina HiSeq 2500	Nipponbare	-
SRP083700	SRR4098173	Root	Unstressed-2 weeks old seedling	Illumina HiSeq 2000	japonica rice	Yuan et al. [16]
SRP083700	SRR4098191	Root	Salt (300 mM NaCl for 12 h)-2 weeks old seedling	Illumina HiSeq 2000	japonica rice	Yuan et al. [16]
SRP114666	SRR5889379	Root	Unstressed-seedling	Illumina HiSeq 2500	Sea Rice 86	Chen et al. [17]
SRP114666	SRR5889380	Root	Unstressed-seedling	Illumina HiSeq 2500	Sea Rice 86	Chen et al. [17]
SRP114666	SRR5889381	Root	Unstressed-seedling	Illumina HiSeq 2500	Sea Rice 86	Chen et al. [17]
SRP114666	SRR5889382	Root	Salt (sea water for 30 d)-seedling	Illumina HiSeq 2500	Sea Rice 86	Chen et al. [17]
SRP114666	SRR5889383	Root	Salt (sea water for 30 d)-seedling	Illumina HiSeq 2500	Sea Rice 86	Chen et al. [17]
SRP114666	SRR5889384	Root	Salt (sea water for 30 d)-seedling	Illumina HiSeq 2500	Sea Rice 86	Chen et al. [17]

**Table 2:** The hub genes detected in the three WGCNA modules.

Modules	Gene_id	Arabidopsis Orthologs	Predicted Functions
Blue	*LOC_Os03g15270*	*AT5G16080*	Carboxyesterase 17
	*LOC_Os05g27340*	*AT5G01750*	Unknown
	*LOC_Os01g72009*	*AT5G04080*	Unknown
	*LOC_Os07g48710*	*AT2G41010*	Calmodulin (CAM)-binding protein of 25 kDa
	*LOC_Os02g13800*	*AT3G24520*	Heat shock transcription factor C1
	*LOC_Os02g43770*	*AT3G60320*	Protein of unknown function (DUF630 and DUF632)
Grey	*LOC_Os02g51080*	*AT1G74470*	Pyridine nucleotide-disulphide oxidoreductase family protein
	*LOC_Os03g20700*	*AT5G13630*	Magnesium-chelatase subunit chlH, chloroplast, putative/Mg-protoporphyrin IX chelatase,
	*LOC_Os01g17170*	*AT3G56940*	Dicarboxylate diiron protein CRD1
	*LOC_Os09g20240*	*AT1G78020*	Unknown DUF581
Turquoise	*LOC_Os03g42520*	*AT1G07985.1*	Expressed protein
	*LOC_Os03g60260*	*AT1G07985.1*	Aromatic and neutral transporter 1
	*LOC_Os03g17790*	*AT2G38905.1*	Low temperature and salt responsive protein family
	*LOC_Os04g48270*	*AT3G61060.1*	Phloem protein PP2-A13
	*LOC_Os08g23870*	*AT5G06760.1*	Late Embryogenesis Abundant 4-5, LEA4-5

## Supplementary Materials

The following are available online at https://www.mdpi.com/2073-4425/10/9/719/s1, Table S1. List of the primers used for the qRT-PCR experiment, Table S2. List of the differentially expressed genes identified in the three datasets, Table S3. Core salt responsive genes in rice classified into three co-expressed modules, Figure S1. Gene ontology (A) and KEGG analysis (B) of DEGs belonging to blue module under salt stress in rice, Figure S2. Gene ontology (A) and KEGG enrichment analysis (B) of DEGs belonging to grey module under salt stress in rice, Figure S3. Gene ontology (A) and KEGG enrichment analyses (B) of DEGs belonging to turquoise module under salt stress in rice.
