# Supplementary figures and images for: WGCNA Analysis of Salt-Responsive Core Transcriptome Identifies Novel Hub Genes in Rice
Source: Genes (Basel). 2019 Sep 17;10(9):719. doi: 10.3390/genes10090719 (PMC6771013; doi:10.3390/genes10090719)

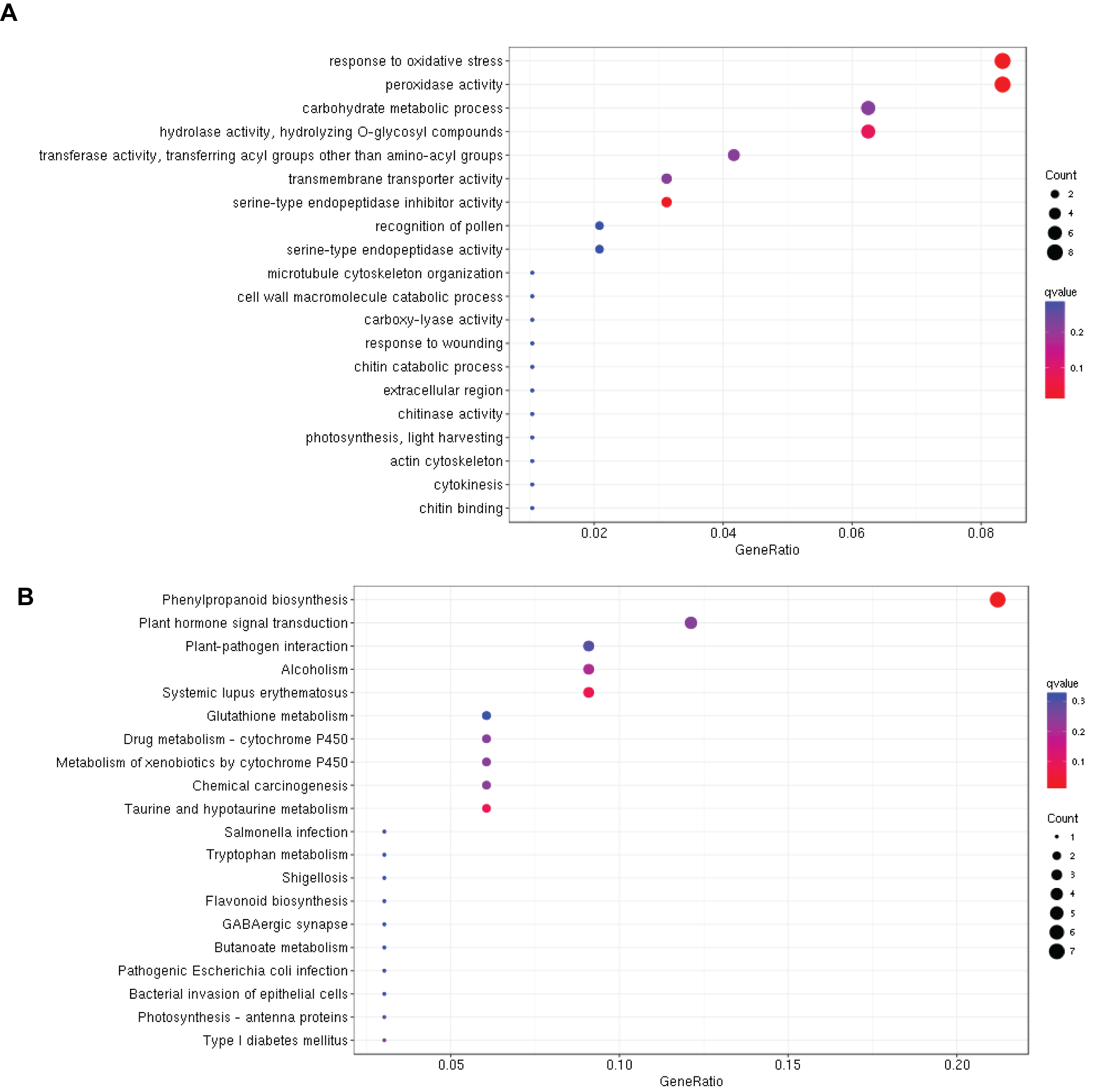

Supplement: Supplementary file 1 [file genes-10-00719-s001.zip › Figure S1.tif]

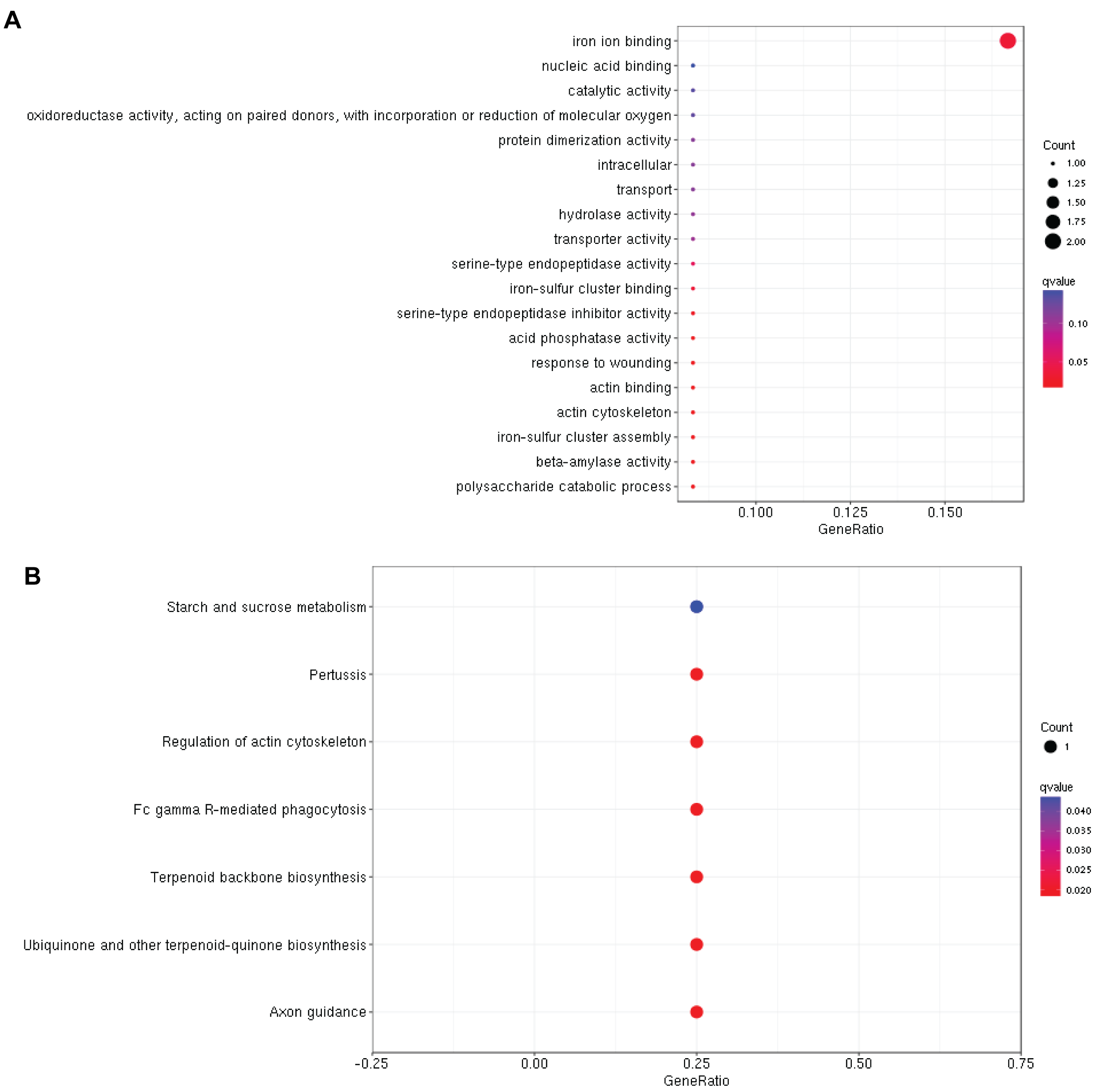

Supplement: Supplementary file 1 [file genes-10-00719-s001.zip › Figure S2.tif]

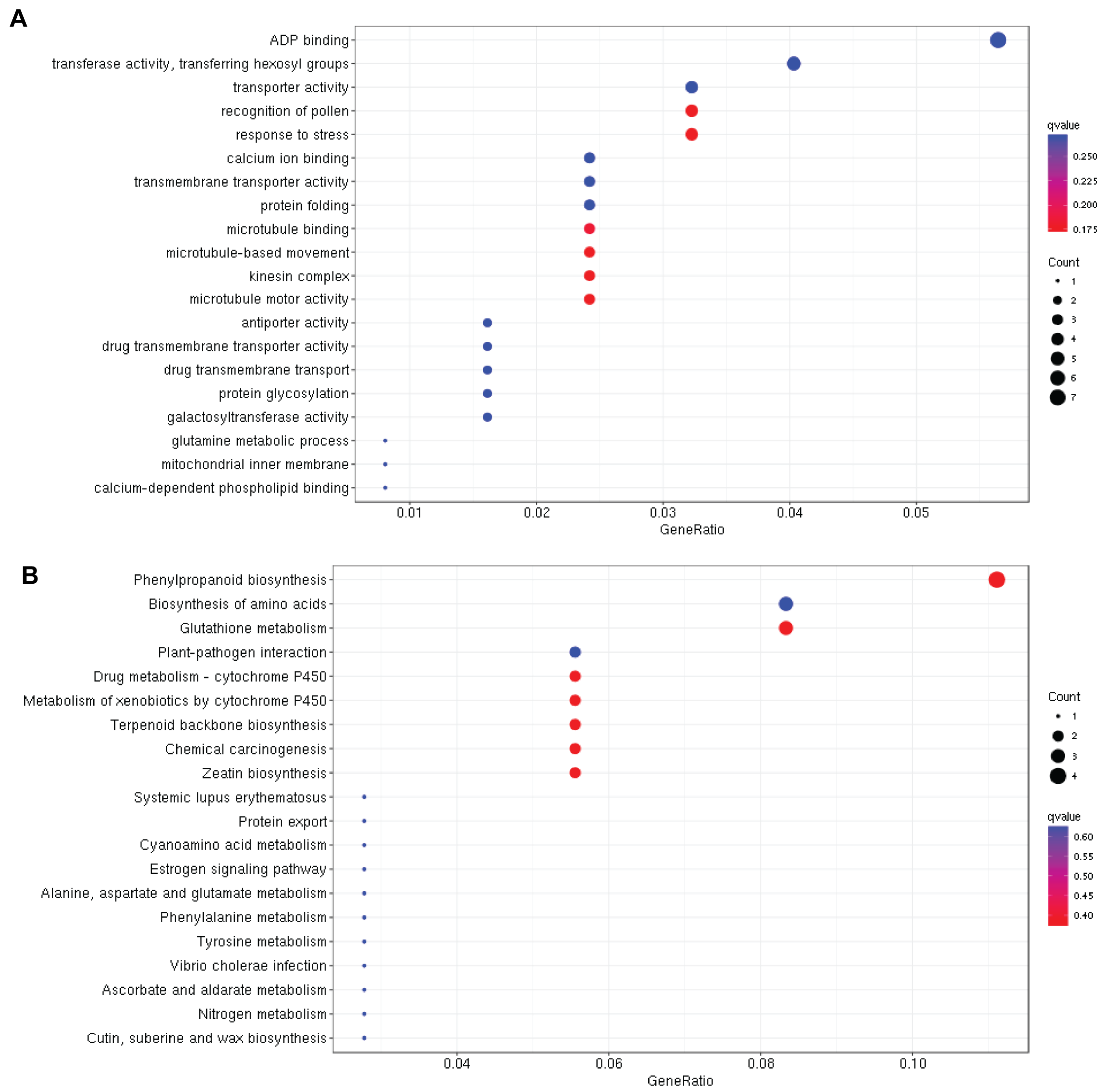

Supplement: Supplementary file 1 [file genes-10-00719-s001.zip › Figure S3.tif]
